# Bronchoalveolar Lavage Fluid Cytology in Healthy Cynomolgus Macaques

**DOI:** 10.3389/fvets.2021.679248

**Published:** 2021-05-25

**Authors:** Vincent Pavot, Christine Prost, Guillaume Dubost-Martin, Kevin Thibault-Duprey, Eve Ramery

**Affiliations:** ^1^Sanofi Pasteur, Research & Development Department, Marcy L'Etoile, France; ^2^Laboratoire de Biologie Clinique, VetAgro-Sup, Campus vétérinaire Marcy l'Etoile, Marcy L'Etoile, France

**Keywords:** bronchoalveolar lavage, BAL, non-human primate, cytology, mucosal immunity, animal models

## Abstract

Bronchoalveolar lavage, or BAL, is a minimally invasive procedure frequently used for clinical and non-clinical research, allowing studies of the respiratory system. Macaques are the most widely used non-human primate models in biomedical research. However, very little information is available in the literature concerning BAL cytology in macaques. The purpose of this study was to establish BAL reference values and document an atlas of BAL cytology from healthy cynomolgus macaques. BALs were obtained from 30 macaques and BAL fluid differential cell counts based on 400 nucleated cells/BAL sample were performed by a board-certified clinical pathologist. Results were analyzed using Reference Value Advisor macroinstructions and the effect of blood and oropharyngeal contaminations was investigated. Overall, nucleated cells interval percentages in BAL fluids were 55.8 to 93.7 for macrophages, 1.8 to 37.1 for lymphocytes, 0.4 to 8.7 for neutrophils, and 0.4 to 9.8 for eosinophils. Mild oropharyngeal contamination did not affect BAL differential cell counts, whilst a slight but significant increase of the percentage of lymphocytes was observed in samples with mild blood contamination. Mucus and variable numbers of ciliated epithelial cells were commonly present. Rarely, multinucleated macrophages and mastocytes were also observed. The reference intervals established in this study provide a useful baseline for the assessment of BAL cytological data in cynomolgus macaques.

## Introduction

Although it does require anesthesia, with the associated risks in respiratory-compromised patients, bronchoalveolar lavage (BAL) is a minimally-invasive and well-tolerated procedure that lessen the need to proceed to more invasive interventions, such as surgical lung biopsy. BAL is applied to the clinical evaluation of patients with various pulmonary disorders, especially interstitial lung disorders, and investigation of bronchial and alveolar diseases (1, 2).

Viral- and bacterial-induced inflammatory diseases of the airways and lungs can cause severe respiratory issues. The development of animal models is of utmost importance to better understand infections and inflammation, and to test the efficacy of vaccines and drugs against those pathogens.

One of the most important, and in demand, animals to be used as models are non-human primates (3). However, only limited information about BAL cytologic reference ranges in cynomolgus macaques is available. Related literature available are based on studies conducted on 5–20 macaques and provided various results because the studies' conditions were different (4–7).

The purpose of the present study was therefore to establish further reference values and to document an atlas from healthy cynomolgus macaques to provide a practical base for further research on respiratory tract disorders and related BAL modifications. This would include understanding the inflammatory response upon exposure to a respiratory pathogen, as the increase of certain cell types in BAL can predict the severity of the disease or the clearance of the pathogen, or even the risk of disease enhancement after vaccination (e.g., Respiratory Syncytial Virus, Coronavirus, etc.) (8–10).

## Materials and Methods

### Animals and Ethics Statement

Thirty, three-years-old, female cynomolgus macaques (*Macaca fascicularis* – Noveprim - Mauritius) 2.7 to 5 kg, were group-housed at Sanofi Pasteur (Marcy l'Etoile – France) in large stainless cages with an automatic watering system and were fed with dry monkey chow (Primate Maintenance Diet 107, SAFE, Route de Saint Bris, 89290 Augy, France) and fresh fruits or vegetables daily. Cynomolgus macaques were provided with behavioral enrichment toys and treats daily. Their health status was determined based on complete physical examination, tuberculin skin tests, serology screening (α-herpes virus, SIV, filovirus, HAV, HBV), fecal examination, and culture for parasites and enterobacteria screening.

The study was reviewed by the Ethics Committee #11 of Sanofi Pasteur and the project has been approved under MESR number APAFIS#5481-2016052710357414 v3. All experiments were conducted following the European Directive 2010/63/UE as published in the French Official Journal of February 7th, 2013.

### Bronchoalveolar Lavages and Cell Staining

Food was removed 12 h prior to anesthesia and BAL procedures. A clinical exam was performed on all cynomolgus macaques to ensure they were healthy. BALs were performed on anesthetized animals with intramuscular injection of Zoletil 50 (tiletamine and zolazepam – Virbac, France) at 3 mg/kg and Rompun 2% (xylazine – Elanco, Germany) at 0.8 mg/kg. Once anesthetized, the macaques were positioned in sternal recumbency on a lifting surgical table. A heating mat was used to limit the hypothermia and one drop of Ocry-gel (carbopol – TVM, France) was applied to the cornea of each eye to prevent dryness. A laryngoscope Miller (Alcyon, France – reference 8017974) was used to visualize the epiglottis, then Xylocaine 5% (lidocaine – Aspen France) was sprayed for local anesthesia. The tongue was gently extended outward with soft pliers after the local anesthetic was allowed to take effect. Macaques were intubated using an uncuffed endotracheal tube with a diameter of 3.5 mm (Alcyon, France – reference 8362375). A silicon spray (Alcyon France – reference 8040708) was used to lubricate the endotracheal tube. Then, a collection catheter (40 cm long, Vygon, France - reference 6281365) was passed down the endotracheal tube to infuse a solution of 5 mL/kg of isotonic sodium chloride NaCl 0.9% at 37°C, based on internal experience and previously published BAL procedure (7). The infusion and the collection were done using a syringe of 50 mL (BD Plastipak, Becton, Dickinson and Company, USA New Jersey), and about half of the solution was recovered. The duration of the procedure between the anesthesia and the collection was ~15 min. Right after the collection, the catheter was pulled off and the macaques were placed under the surveillance of the clinical veterinarian on a recovery table also equipped with heat mats. The endotracheal tube was connected to an oxygen supply, oxygenation, and cardiac rhythm were monitored with a PM60VET oximeter (Mindray Medical – reference 8288905 at Alcyon, France). An average of 15 min was necessary until oxygen and heart rate returned to normality (>95% of oxygen and ~100 beats per minute). For a faster awakening, 0.04 mL/kg of Antisedan (atipamezole – Orion corporation, Finland) was injected into the animals by intramuscular route. No untoward effect was noted during or following fluid instillation and the macaques woke up normally after 1 h.

BAL fluids were collected in 15 mL-tubes (Falcon) and were placed at 4°C until staining (less than 1 h). Tubes were centrifuged at 400 x g for 7 min at 4°C to pellet cells and mucus. BAL pellets were resuspended with 20 μL of PBS to prepare smear slides. Slides were stained with a commercially available modified Giemsa stain (Differential Quik Stain Kit - Polysciences). Following the coloration steps, the slides were rinsed in distilled water, quickly dehydrated in 100% EtOH, and dipped in methylcyclohexane before being mounted with a synthetic resin.

### Cytology Analysis

BAL differential cell counts were performed on a total of 400 nucleated cells per sample (1 BAL sample/macaque) by a board-certified clinical pathologist. By convention, this does not include epithelial cells. The results of the differential cell counts were expressed as a percentage composition.

Histograms of all results were visually analyzed to detect possible outliers. As the Clinical and Laboratory Standards Institute recommends, the values were retained rather than deleted unless they were known to be aberrant observations (11). Normality of the distribution of native or transformed values was tested using the Anderson–Darling test. Finally, reference intervals and 90% confidence intervals (CI) of the limits were determined using the Reference Value Advisor macroinstructions (12) for Excel (Microsoft Corp., Redmond, WA, USA), with the guidance of American Society for Veterinary Clinical Pathology reference interval guidelines (13). As the sample size was too small (*n* <40) to compute a non-parametric reference interval, the robust method with a Box-Cox transformation was used after checking the symmetry of the distribution. The effect of blood and oropharyngeal contamination was tested using GraphPad Prism 8.0 (GraphPad Software Inc., La Jolla, USA) and statistical significance was set at *P* < 0.05. Unpaired *T*-test assuming Gaussian distribution was performed.

## Results

### Bronchoalveolar Lavage Cytology

Descriptive statistics of BAL differential cell counts (mean, SD, minimum, maximum), obtained from non-transformed data, and reference interval with their 90% confidence intervals (CI), obtained by robust method after Box-Cox transformation, are provided in Table 1. No outlier was rejected because none was outside the median ± the interquartile range (between the 75th and 25th percentiles).

**Table 1:** Descriptive statistics and reference intervals of differential cell counts in bronchoalveolar lavage (BAL) fluid in 3-years-old healthy macaques.

	**Macrophages**	**Lymphocytes**	**Neutrophils**	**Eosinophils**
Number of macaques	30	30	30	30
Mean (%)	76.0	16.8	4.3	2.7
Median (%)	75.5	15.4	4.0	2.0
SD	9.1	8.6	2.0	2.1
Minimum (%)	54.7	1	0.3	0.3
Maximum (%)	95.7	37.3	8	8.0
Lower limit of reference interval (%)	55.8	1.8	0.4	0.4
Upper limit of reference interval (%)	93.7	37.1	8.7	9.8
90% CI for lower limit	50.1	0.2	ND	0.2
	61.9	4.7	1.2	0.5
90% CI for upper limit	89.2	30.4	7.7	6.2
	97.6	43.6	9.9	14.1

*ND, Not determined; CI, confidence interval; SD, standard deviation*.

**Figure 1:**
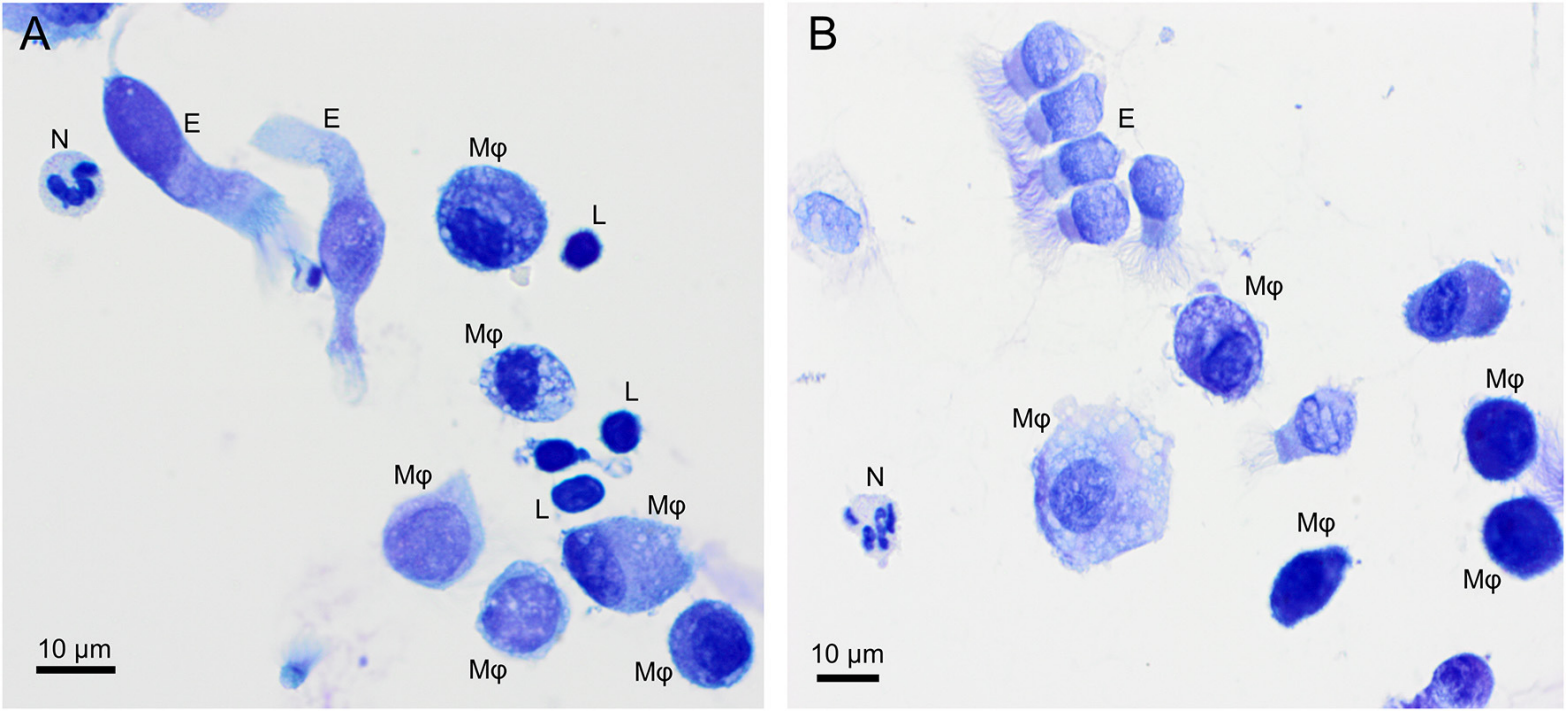
Overview of cells found in a bronchoalveolar lavage sample from two healthy cynomolgus macaques. **(A)** macaque #1, **(B)** macaque #2. Alveolar macrophages (Mφ) predominate, followed by lymphocytes (L). Neutrophil (N) and occasional ciliated columnar epithelial cells (E) are also present. Modified Giemsa. X50 objective. Oil immersion.

Alveolar macrophage was the predominant cell type to be identified in the lavage fluid samples (Figure 1), with a reference interval percentage between 55.8 and 93.7%. Lymphocytes were comprised between 1.8 and 37.1%, neutrophils between 0.4 and 8.7%, and eosinophils between 0.4 and 9.8% (Table 1).

**Figure 2:**
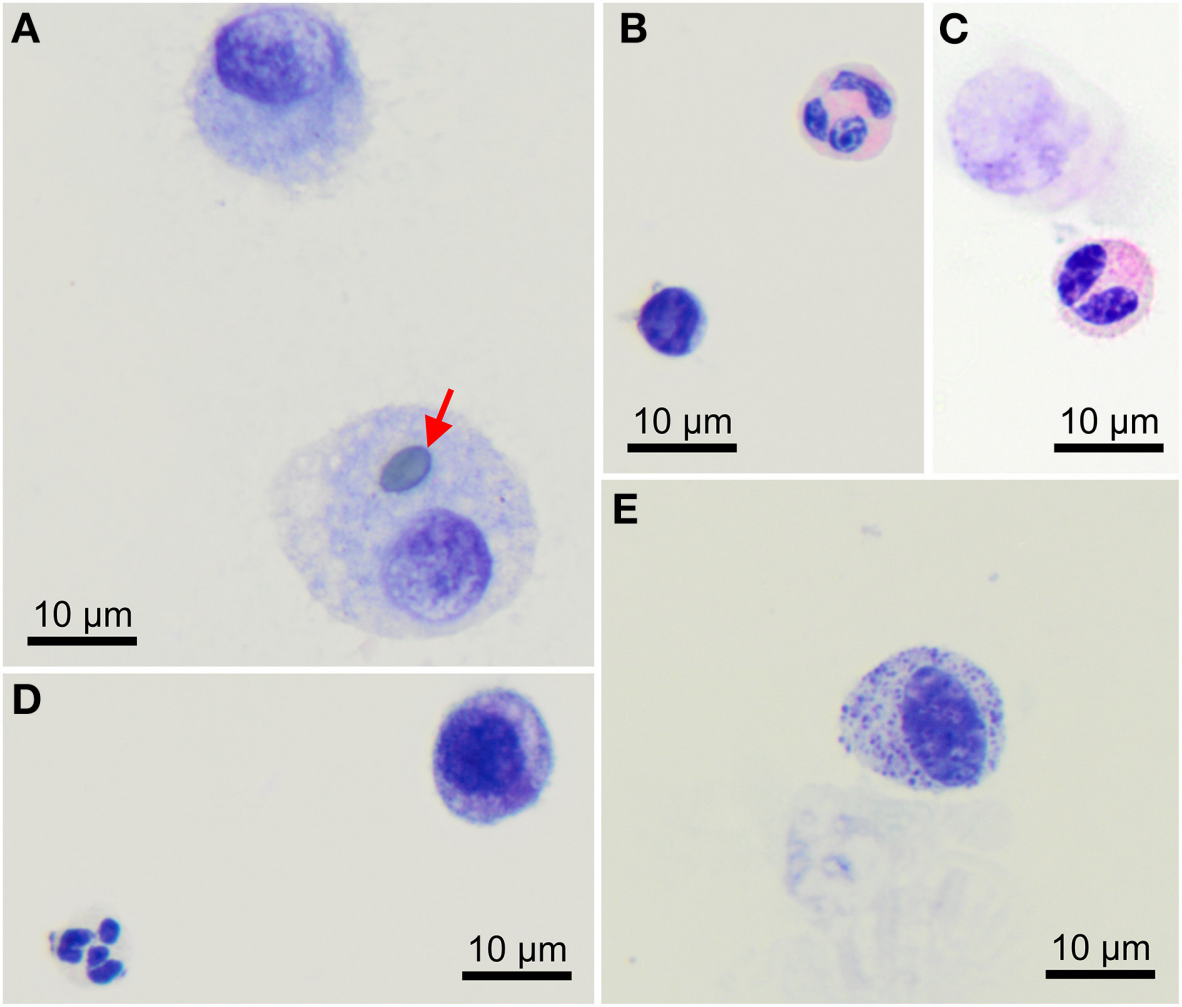
Morphology of cells found in bronchoalveolar lavage samples from healthy cynomolgus macaques. **(A)** Macrophages, one of them contains a pollen grain (arrow). **(B)** One lymphocyte (bottom left) and one eosinophil (upper right). **(C)** Bilobed eosinophil (bottom). **(D)** One well-preserved neutrophil (bottom left) and a macrophage (upper right). **(E)** Mast cell. Modified Giemsa. X100 objective. Oil immersion.

Macrophages are large roundish cells occurring singly (Figures 1, 2A). Their nuclei can be round, ovoid, or bean-shaped and located anywhere in the cell. The cytoplasm is abundant, moderately basophilic, frequently foamy to vacuolated, and occasionally contains cellular and environmental debris. Rare multinucleated macrophages may be seen.

Lymphocytes (Figures 1A, 2B) were the second most represented population. They are small round cells with a high nucleocytoplasmic ratio. The nucleus contains dense chromatin. The cytoplasm is scant and usually reduced to a one-side crescent. However, it is important to check for the presence of cytoplasm to avoid counting naked nuclei as lymphocytes.

Neutrophils (Figures 1, 2D) and eosinophils (Figures 2B,C) may be difficult to differentiate in macaques, as neutrophils contain pale pink granules in their cytoplasm. However, eosinophils have more distinct, roundish, and brighter pink granules. Also, neutrophils usually exhibit multiple segmentation whereas eosinophils are more often bilobed. Rare unsegmented eosinophils were also observed.

Variable numbers of columnar epithelial cells of the respiratory tract were present on all slides and are part of the normal collected cells (Figure 1). They occur singly or in palisading arrangements. They have a round, basal nucleus, with fine chromatin. Their cytoplasm is lightly basophilic and, when well-preserved, cilia can be observed on the apical border.

A single macaque exhibited a single mastocyte in its BAL (Figure 2E). Mastocytes are round cells with an unsegmented, round to ovoid nucleus. The cytoplasm is moderately abundant and filled with azurophilic granules. With May-Grünwald Giemsa staining, the granules frequently obscure the nucleus. With the modified Giemsa stain, granules typically appear faint. This data was not included in statistics.

**Figure 3:**
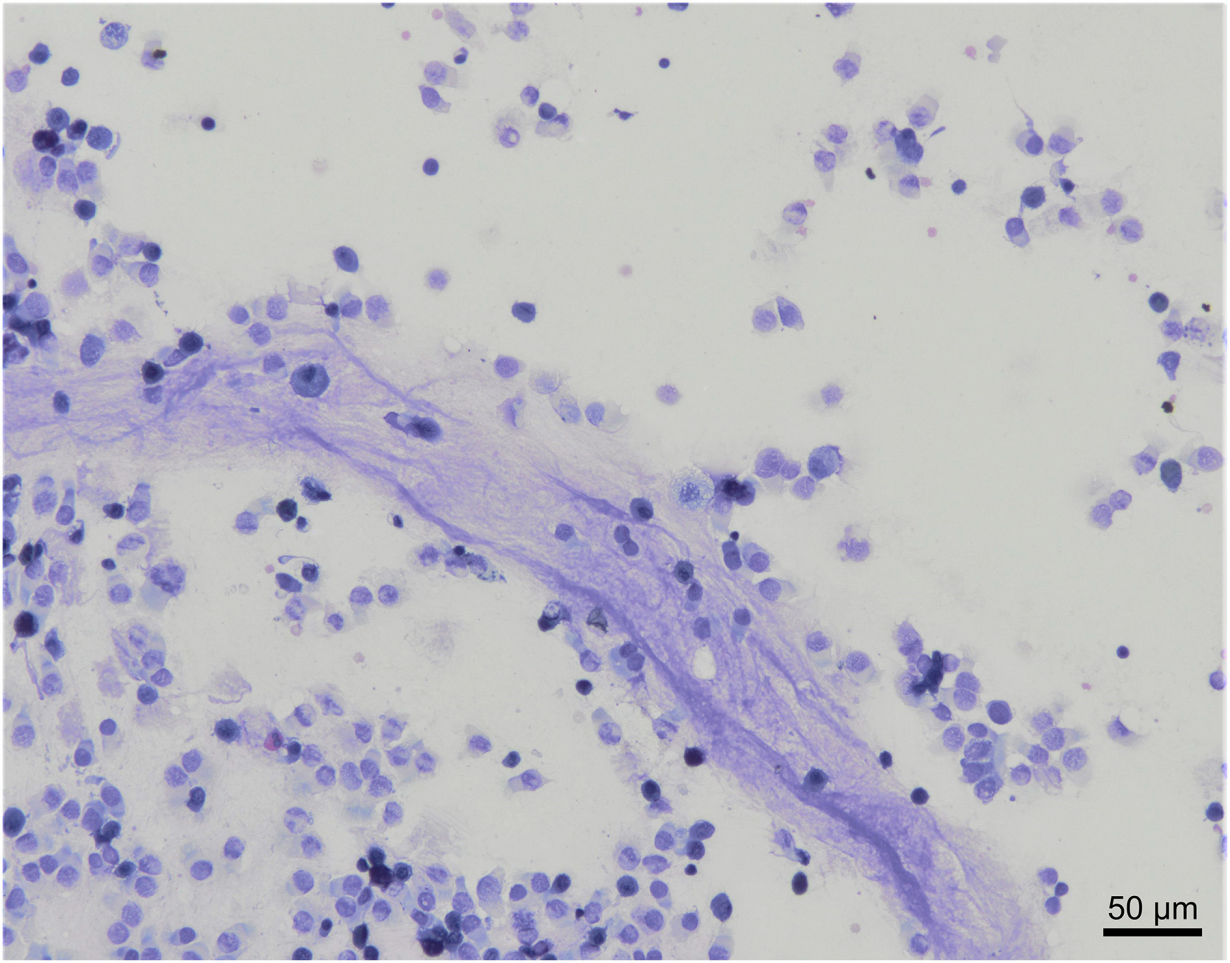
Mucus observation in a bronchoalveolar lavage cytology from a healthy cynomolgus macaque. Low amount of mucus is admixed with nucleated cells and columnar epithelial cells. Modified Giemsa. X20 objective.

A low amount of mucus was consistently observed as a cloudy network of pale blue to purple material (Figure 3).

### Influence of Blood and Oropharyngeal Contamination on BAL Cytology

**Figure 4:**
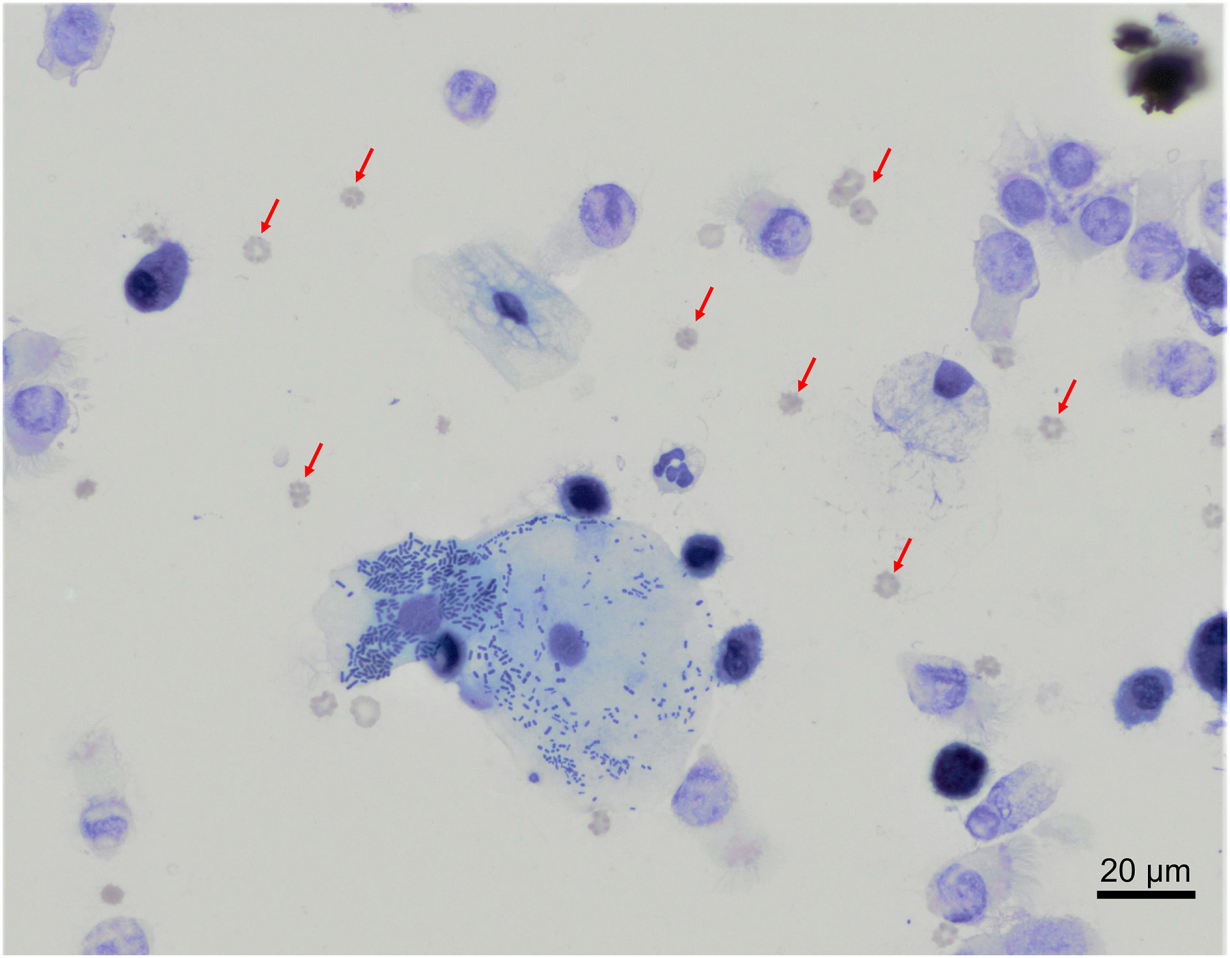
Mixed contaminations of a bronchoalveolar lavage from a healthy cynomolgus macaque. Several erythrocytes (red arrows) are present on the background. In the center, a large squamous epithelial cell exhibits multiple rode-shaped bacteria on its surface. Modified Giemsa. X50 objective. Oil immersion.

**Figure 5:**
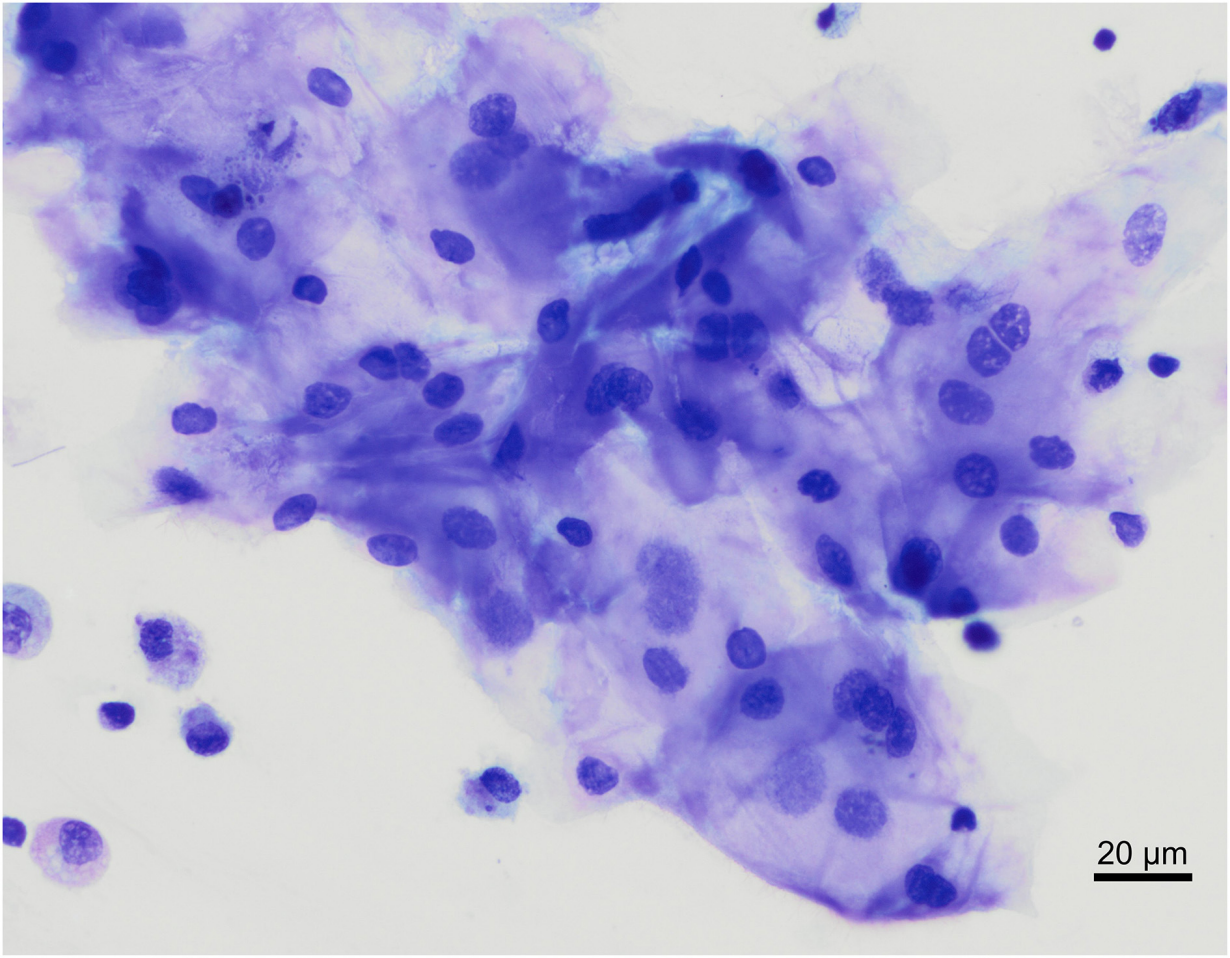
Oropharyngeal contamination of a bronchoalveolar lavage from a healthy cynomolgus macaque. Raft of squamous epithelial cells located in the middle of the image, with low numbers of macrophages and small lymphocytes on the bottom left corner. Modified Giemsa. X50 objective. Oil immersion.

**Figure 6:**
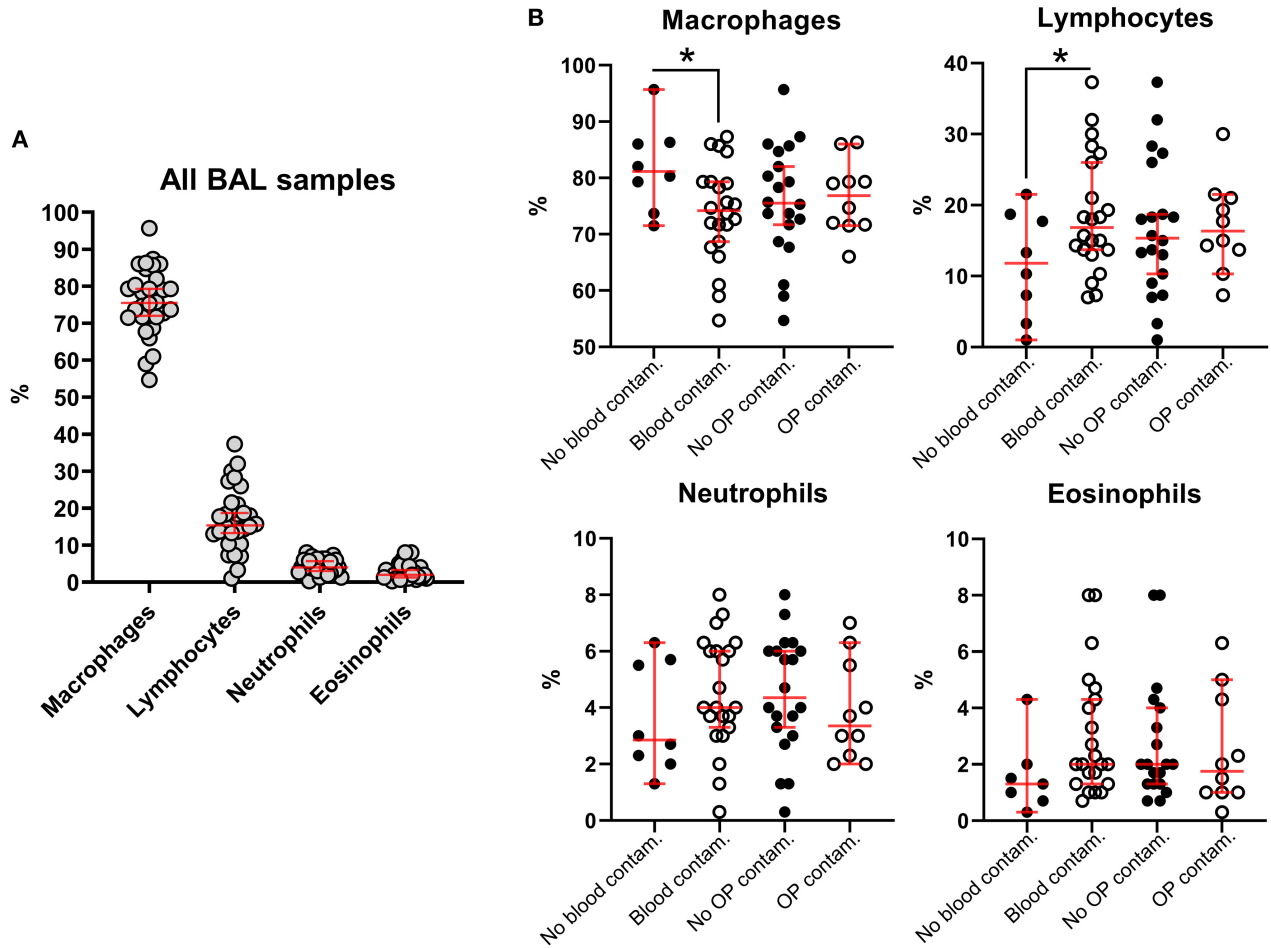
Influence of blood and oropharyngeal contamination on percentages of the different nucleated cell subsets in BALs from healthy macaques. **(A)** All BAL samples, regardless of blood or oropharyngeal contamination. **(B)** Cell subsets considering blood and oropharyngeal contaminations. Each dot is representative of one specimen. Bars, medians ± 95% confidence interval; OP, oropharyngeal; Contam., contamination. *P-value < 0.05.

In our study, 22 out of 30 BAL samples revealed mild to moderate blood contamination (<10 red blood cells/field, 100X objective, oil immersion – Figure 4) and 10 out of 30 had mild oropharyngeal contamination (Figure 5). Oropharyngeal contamination is recognized by the presence of squamous epithelial cells. These cells are large, angular, with a small ovoid nucleus, centrally located in the cell. Chromatin is dense and devoid of nuclei. The cytoplasm is abundant and can be transparent, light pink to blue depending on the stain. Numerous bacteria can be present at the surface of the cell, originating from the “normal” microbiota of the oral cavity (Figure 4). Bacteria from oropharyngeal contamination are extracellular and do not impact the differential cell counts. Oropharyngeal contamination had no statistical effect on differential cell counts (Figure 6). A slightly yet significant higher percentage of lymphocytes (*P* < 0.05) was observed in samples with mild blood contamination—median without blood contamination: 12% [95% CI, 1–21%]; median with blood contamination: 17% [95% CI, 13–26%]—with a concurrent slight decrease in the percentage of macrophages (*P* <0.05). Neutrophils and eosinophils were not statistically affected.

## Discussion

This study proposes reference intervals for BAL fluid differential cell counts, generated from 30 healthy female cynomolgus macaques. Alveolar macrophage was the predominant cell type to be identified, with a reference interval percentage between 55.8 and 93.7%. Lymphocytes were comprised between 1.8 and 37.1%, neutrophils between 0.4 and 8.7%, and eosinophils between 0.4 and 9.8%.

Among eosinophils, we observed unsegmented eosinophils that may represent immature eosinophils or a subset of homeostatic eosinophils. Indeed, eosinophils have been perceived for a long time as terminally differentiated cytotoxic and destructive cells but recent studies have demonstrated that eosinophils also exert a variety of essential homeostatic functions and display unique morphological and phenotypical features that unambiguously distinguish them from the inflammatory eosinophils, especially in the lungs (14, 15).

Although May-Grünwald Giemsa is considered the gold standard in cytology evaluation, modified Giemsa fast staining (Diff-Quick) was used in this study as more representative of daily practice in research labs. The same staining protocol was used in previous studies (4–6).

The results obtained in our study are comparable to results published in other species, including humans (16), horses (17), dogs and cats (18). Only a few previously published papers provide some references concerning BAL cytology in different macaque species (4–7). They were established from 5 to 20 individuals and reported as mean or median ± standard deviation (SD). The present study complies with the recently published recommendations concerning small reference sample groups (19), resulting in much wider reference intervals. Conflicting results concerning macrophages were previously published as Tate et al. (5) reported a high percentage of macrophages of 98.33 ± 1.14 (mean ± SD) whilst others obtained results comparable to ours (4, 6, 7). Also, we obtained slightly higher percentages of lymphocytes. This discrepancy may be related to breeding conditions or explained by several differences during BAL collection and processing. Collection by video-endoscopy vs. endotracheal tube could have resulted in the collection of different segments of the lung with slightly different populations of cells. In our study, processing was kept to a minimum: BALs were centrifuged, and the pellets were directly smeared on slides. In previous studies (4–6), macaques were of different origin, sample collection was slightly different with some performing lavages at necropsy or performing BAL cells centrifugation and resuspension steps and sometimes hemolysis, that may have resulted in an alteration from the original cell population. Also, in our study, the slight blood contamination could be responsible for a small overestimation of lymphocytes as those cells are one of the predominant leukocyte population in peripheral blood. Indeed, from the author's experience, rare red blood cell and oropharyngeal contaminants are frequently found in BAL from healthy mammals (dogs, cats, and horses) and are technique-dependent. Oropharyngeal and slight blood contamination may result from slight scratches of the oropharyngeal and tracheobronchial walls by the endoscope or the endotracheal tube. Hemorrhage can also occur secondary to depression during aspiration of the fluid especially if a small airway is sampled. Although blood contamination was not reported in the other studies (4–7), more contamination is expected with the endotracheal tube as this procedure is blinded. Yet, this technique is frequently used in practice as fiberoptic endoscope is not always available in facilities.

In the present study, we took advantage of the presence of both, non-contaminated and slightly contaminated samples (blood and oropharyngeal contaminants)—which from our experience reflects reality—to evaluate the impact of such contaminations on differential cell counts. As expected, our results provide further evidence that these minor contaminations do not impact differential results in an extent that would alter clinical interpretation. Still, statistically significant differences were observed in macrophage and lymphocyte percentages with the presence or absence of blood. Because lymphocytes are the predominant nucleated blood cells in macaques, the higher lymphocyte percentage in blood-contaminated samples was primarily attributed to the blood contamination. However, subtle lymphocytic inflammation, resulting in increased fragility of capillaries and increased susceptibility for hemorrhage, cannot be formally excluded. We believe that it is preferable not to hemolyze to assess the extent of blood contamination.

To further investigate BALs, it would be interesting to measure the total number of nucleated cells in the BAL. Although this number varies with the method used, it remains a good indicator of inflammation or of any changes in BAL.

Of note, the homogeneity of the macaque population investigated in our study could introduce a bias. Indeed, the reference sample group is composed exclusively of females of approximately the same age. Females are easier to handle and as such represent a significant subset in preclinical studies. We, therefore, considered our macaque population as representative of middle-age specimens investigated in most preclinical studies. However, a specific study would be interesting to investigate the effect of gender on BAL composition.

## Conclusion

This study provides a useful baseline for the assessment of BAL cytological data in cynomolgus macaques. Before differential cell count, it is advised to assess the sample for the presence of blood and oropharyngeal contamination. This study also indicates that a few superficial squamous cells and up to 10 RBCs/field at objective 100X can be tolerated.

## Data Availability Statement

The raw data supporting the conclusions of this article will be made available by the authors, without undue reservation.

## Ethics Statement

The study was reviewed by the Ethics Committee #11 of Sanofi Pasteur and the project has been approved under MESR number APAFIS#5481-2016052710357414 v3.

## Author Contributions

VP, CP, ER, and KT-D designed and supervised the research. GD-M and ER performed the experiments. ER and VP provided a first draft of the manuscript. All authors were involved in the analysis and/or interpretation of the data, drafting or critically revising the manuscript, approved the final version and are accountable for the accuracy, and integrity of the manuscript.

## Conflict of Interest

VP, CP, GD-M, and KT-D were all employees of Sanofi Pasteur at the time of this study and hold company stocks. The remaining author declares that the research was conducted in the absence of any commercial or financial relationships that could be construed as a potential conflict of interest.
